# Supplementary material for: Resolving the full spectrum of human genome variation using Linked-Reads
Source: Genome Res. 2019 Apr;29(4):635–45. doi: 10.1101/gr.234443.118 (PMC6442396; doi:10.1101/gr.234443.118)
Supplement: Supplemental Material [file supp_gr.234443.118_Supplemental_longranger-2.2.zip › longranger-2.2.tar.bz2!/lr2.2-public/lib/stan/cmdstan/stan/doc/api/html/dir_bfdd916fd7a3310c7c8c32c9dbb4c5dd.html]

Stan: src/stan/gm/grammars Directory Reference


|  |  |
| --- | --- |
|  | Stan  2.5.0  probability, sampling & optimization |


- Main Page
- Related Pages
- Namespaces
- Classes
- Files

All Classes Namespaces Files Functions Variables Typedefs Enumerations Enumerator Friends Macros Pages

- src
- stan
- gm
- grammars

grammars Directory Reference

|  |  |
| --- | --- |
| Files | |
| file | bare\_type\_grammar.hpp [code] |
|  | |
| file | bare\_type\_grammar\_def.hpp [code] |
|  | |
| file | bare\_type\_grammar\_inst.cpp [code] |
|  | |
| file | common\_adaptors\_def.hpp [code] |
|  | |
| file | expression07\_grammar.hpp [code] |
|  | |
| file | expression07\_grammar\_def.hpp [code] |
|  | |
| file | expression07\_grammar\_inst.cpp [code] |
|  | |
| file | expression\_grammar.hpp [code] |
|  | |
| file | expression\_grammar\_def.hpp [code] |
|  | |
| file | expression\_grammar\_inst.cpp [code] |
|  | |
| file | functions\_grammar.hpp [code] |
|  | |
| file | functions\_grammar\_def.hpp [code] |
|  | |
| file | functions\_grammar\_inst.cpp [code] |
|  | |
| file | iterator\_typedefs.hpp [code] |
|  | |
| file | program\_grammar.hpp [code] |
|  | |
| file | program\_grammar\_def.hpp [code] |
|  | |
| file | program\_grammar\_inst.cpp [code] |
|  | |
| file | statement\_2\_grammar.hpp [code] |
|  | |
| file | statement\_2\_grammar\_def.hpp [code] |
|  | |
| file | statement\_2\_grammar\_inst.cpp [code] |
|  | |
| file | statement\_grammar.hpp [code] |
|  | |
| file | statement\_grammar\_def.hpp [code] |
|  | |
| file | statement\_grammar\_inst.cpp [code] |
|  | |
| file | term\_grammar.hpp [code] |
|  | |
| file | term\_grammar\_def.hpp [code] |
|  | |
| file | term\_grammar\_inst.cpp [code] |
|  | |
| file | var\_decls\_grammar.hpp [code] |
|  | |
| file | var\_decls\_grammar\_def.hpp [code] |
|  | |
| file | var\_decls\_grammar\_inst.cpp [code] |
|  | |
| file | whitespace\_grammar.hpp [code] |
|  | |
| file | whitespace\_grammar\_def.hpp [code] |
|  | |
| file | whitespace\_grammar\_inst.cpp [code] |
|  | |


---

[ Stan Home Page ]

*© 2011–2014,
Stan Development Team.*
